# Indoor Pedestrian Positioning Method Based on Ultra-Wideband with a Graph Convolutional Network and Visual Fusion

**DOI:** 10.3390/s24206732

**Published:** 2024-10-19

**Authors:** Huizhen Mu, Chao Yu, Shuna Jiang, Yujing Luo, Kun Zhao, Wen Chen

**Affiliations:** 1 Engineering Center of SHMEC for Space Information and GNSS, East China Normal University, Shanghai 200241, China; ecnu_71255904094@yeah.net (H.M.); 51255904048@stu.ecnu.edu.cn (S.J.); 51255904047@stu.ecnu.edu.cn (Y.L.); kzhao@ce.ecnu.edu.cn (K.Z.); wchen@sist.ecnu.edu.cn (W.C.); 2 Shanghai Key Laboratory of Multidimensional Information Processing, East China Normal University, Shanghai 200241, China; 3 Key Laboratory of Geographic Information Science, Ministry of Education, East China Normal University, Shanghai 200241, China

**Keywords:** indoor fusion positioning, UWB, vision sensor, GCN, particle filter

## Abstract

To address the challenges of low accuracy in indoor positioning caused by factors such as signal interference and visual distortions, this paper proposes a novel method that integrates ultra-wideband (UWB) technology with visual positioning. In the UWB positioning module, the powerful feature-extraction ability of the graph convolutional network (GCN) is used to integrate the features of adjacent positioning points and improve positioning accuracy. In the visual positioning module, the residual results learned from the bidirectional gate recurrent unit (Bi-GRU) network are compensated into the mathematical visual positioning model’s solution results to improve the positioning results’ continuity. Finally, the two positioning coordinates are fused based on particle filter (PF) to obtain the final positioning results and improve the accuracy. The experimental results show that the positioning accuracy of the proposed UWB positioning method based on a GCN is less than 0.72 m in a single UWB positioning, and the positioning accuracy is improved by 55% compared with the Chan–Taylor algorithm. The proposed visual positioning method based on Bi-GRU and residual fitting has a positioning accuracy of 0.42 m, 71% higher than the Zhang Zhengyou visual positioning algorithm. In the fusion experiment, 80% of the positioning accuracy is within 0.24 m, and the maximum error is 0.66 m. Compared with the single UWB and visual positioning, the positioning accuracy is improved by 56% and 52%, respectively, effectively enhancing indoor pedestrian positioning accuracy.

## 1. Introduction

In the era of information technology, the widespread adoption of wireless communication devices has propelled the flourishing of location-based services (LBS) [1]. Typical indoor positioning techniques are Bluetooth positioning [2], Wi-Fi positioning [3], radio frequency identification (RFID) positioning [4], ZigBee positioning [5], and ultra-wideband (UWB) positioning techniques [6]. Among these techniques, the UWB positioning technique has the advantages of solid penetration ability, low transmission power and high transmission rate, and a relatively broad application in indoor positioning [7]. However, the propagation of ultra-wideband signals in indoor environments is susceptible to multipath effects and non-line-of-sight (NLOS) scenarios, which affect positioning accuracy [8]. Concurrently, the evolution of artificial intelligence and deep learning has empowered vision-based target-detection and positioning methods [9,10], which provide cost-effective and scalable alternatives without requiring additional devices to be carried by the target.

Several studies have addressed the architectural challenges of integrating multiple localization systems. Literature [11] introduced a handoff protocol to enable seamless switching between different localization systems, thereby improving reliability when one system fails. Similarly, literature [12] investigated radio-frequency handoff strategies, underscoring the importance of adaptable architectures capable of dynamically adjusting to changing environmental conditions. Literature [13] emphasized the need for reproducibility and transparency in localization research, advocating for standardized methods to compare system performance across studies. Despite these advancements, challenges such as sensor drift, multipath effects, and sensitivity to environmental conditions remain unresolved, especially in complex indoor environments.

Privacy concerns have also emerged with the increased use of indoor localization systems, especially for contact tracing and social distancing during the COVID-19 pandemic [14]. Literature [15] proposed privacy-preserving protocols to address these concerns while maintaining localization accuracy. In addition, literature [16] explored integrating heterogeneous localization systems to create a more unified user experience in location-based services.

Despite these advancements, single-sensor positioning techniques face limitations in dynamic and complex environments. Thus, multi-sensor fusion approaches have gained traction in current research. For instance, literature [17] used an extended particle filter to fuse UWB and IMU data, reducing jitter in positioning results. However, this method requires tight synchronization between UWB tags and IMUs, increasing the cost and complexity of the system. Other studies, such as literature [18,19], have explored the combination of visual and inertial data, but these methods still face challenges, such as high computational requirements and difficulty maintaining continuous positioning over extended periods. Moreover, the fusion of UWB signals with vision-based systems, as explored by R. Zhang, D et al., often suffers from limitations during dynamic positioning, where visual data may become unreliable due to image jitter during movement [20,21].

To address these challenges and improve positioning in NLOS environments, we propose a novel system that fuses UWB and visual positioning based on particle filtering. Our contributions are as follows:

(1) UWB positioning module based on a GCN: We propose an indoor pedestrian positioning method utilizing a GCN. By leveraging the powerful feature-extraction capabilities of GCNs, our method integrates features from neighboring positioning points to improve accuracy, advancing beyond traditional algorithms like the Chan–Taylor method.
(2) Visual localization module based on Bi-GRU: We introduce a visual localization method that employs a Bi-GRU network. This model effectively compensates for visual localization errors caused by camera distortions by learning and correcting residual errors in the positioning results.
(3) Data fusion algorithm based on PF: We developed a particle filter-based fusion algorithm that combines data from UWB and visual systems. By considering the uncertainty and noise from both types of sensors, our fusion algorithm improves the accuracy and robustness of positioning.

By integrating two sensor modalities, our approach mitigates the limitations of single-sensor systems and provides more reliable positioning, even in environments with severe signal degradation due to multipath effects and NLOS conditions.

## 2. UWB Indoor Positioning

In UWB indoor positioning technology, conventional TDOA methods such as the Chan and Taylor algorithms encounter challenges, particularly in environments with significant noise or when the initial position estimates are inaccurate. To address these issues, we propose an improved UWB indoor pedestrian positioning method based on a GCN. The GCN-based method aggregates data from neighboring positioning nodes and can model complex nonlinear relationships in indoor environments, providing a more accurate and robust positioning solution. Finally, we describe the full positioning process using a GCN, including data acquisition, model training, and real-time pedestrian positioning.

### 2.1. UWB Indoor Positioning Method Based on TDOA

Given UWB’s superior temporal resolution, time of arrival (TOA) and TDOA are the prevalent techniques employed for positioning. Compared to TOA, TDOA can reduce the dependence on time synchronization and has a lower cost. Therefore, this work uses the TDOA data for positioning.

Classical TDOA-solving methods include the Chan and Taylor algorithms [22,23]. The Chan algorithm requires minor errors in the TDOA measurements, and the noise is a zero-mean Gaussian distribution. The positioning performance degrades faster when significant deviations in the geometric position exist. The Taylor algorithm relies more on the initial position estimation. When the initial value is inaccurate, its computation converges slowly and may even diverge, thus obtaining a poor approximate solution [24].

### 2.2. UWB Indoor Positioning Method Based on a GCN

To solve the issues with conventional TDOA methods, we propose an UWB indoor pedestrian positioning method based on a GCN. GCNs can aggregate data from neighboring nodes and uncover complex nonlinear relationships between node features and pedestrian locations, improving positioning accuracy in indoor environments.

In our experiment, the positioning trajectory of a pedestrian is represented as a graph where each node corresponds to a specific time point in the pedestrian’s movement trajectory. We selected 15 nodes to represent temporally consecutive positioning points in each graph, thus constructing a temporal sequence of movements.

The adjacency matrix ***A***, which defines the connectivity between nodes, is a square matrix with dimensions of 15 × 15. This matrix encodes the relationships between consecutive nodes in time, with each node connected to its previous and subsequent nodes to maintain the spatial–temporal relationships between the pedestrian’s positioning points. The features of each node include the 3D coordinates of the four UWB base stations and the corresponding TDOA data, resulting in a feature vector of 15 dimensions.

**Figure 1:**
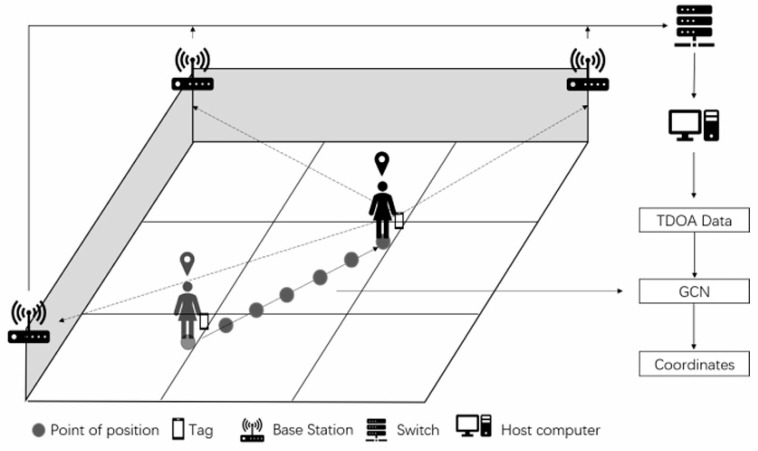
The indoor pedestrian positioning schematic.

**Figure 2:**
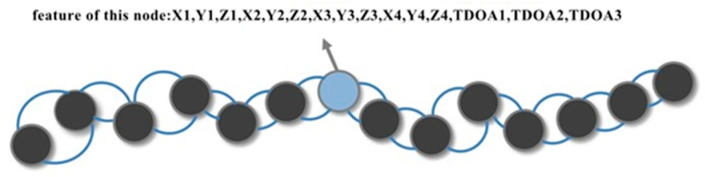
Building the pedestrian trajectory positioning points into the form.

To apply graph convolutional neural networks to indoor positioning information solving, the first step is to construct a data format that conforms to the inputs of a GCN. The indoor pedestrian positioning schematic and the process of building the pedestrian trajectory positioning points into the form of graph data are shown in Figure 1 and Figure 2.

**Figure 3:**
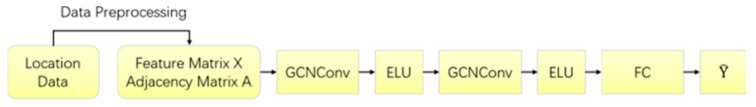
The graph convolutional neural network model for UWB positioning.

As shown in Figure 1, pedestrians’ front and back locations have a naturally spatiotemporal relationship. Therefore, the indoor pedestrian movement trajectory can be constructed into graph data that conform to the input of the graph neural network. A positioning point is represented as a node of the graph, and the data features of the positioning point are the nodes’ features. Positioning points generated at two adjacent moments are connected by an edge. The features of each node include the 3D coordinate information of the four base stations and TDOA data generated by the four base stations—a total of (3 × 4 + 3 = 15) features. Thus, the features of one node can be expressed as in Figure 2. By selecting m position nodes that are temporally continuous in the pedestrian’s moving trajectory, the input to the graph convolutional neural network is a matrix with dimensions of m × 15. We consider the position coordinates of two neighboring moments the closest when the pedestrian moves. Therefore, the adjacency matrix is represented as the positioning point of a particular moment that is only connected to its previous and subsequent positioning points by edges. The graph convolutional neural network model for UWB positioning constructed in this paper is shown in Figure 3.

The network model consists of three main modules:

(1) Input Module. The inputs are the coordinates of the four base stations, and the positioning data consist of three TDOA values. By selecting m localized nodes that are consecutive in the time series, the feature matrix of the input graph convolutional neural network is an m × 15-dimensional matrix. Thus, the input data are defined as follows:
  (1) $$\left[\begin{array}{ccccccc} C_{1} & C_{2} & C_{3} & C_{4} & TDOA_{1}^{1} & TDOA_{2}^{1} & TDOA_{3}^{1}\\ C_{1} & C_{2} & C_{3} & C_{4} & TDOA_{1}^{2} & TDOA_{2}^{2} & TDOA_{3}^{2}\\ & & & & \ldots & & \\ C_{1} & C_{2} & C_{3} & C_{4} & TDOA_{1}^{m} & TDOA_{2}^{m} & TDOA_{3}^{m} \end{array}\right]$$
  where *C_v_* = (*X_v_*, *Y_v_*, *Z_v_*) (*v* = 1, 2, 3, 4) denotes the coordinates of the *v*th UWB base station, and $TDOA_{i}^{m}$ denotes the TDOA value of the *m*th localized point to the host station and the *i*th slave base station. We used three slave base stations, so *i* = 1, 2, 3.
  For a graph with m nodes, its adjacency matrix ***A*** ∈ R^m^^*m^ is denoted:
  (2) $$A_{ij}=\left\{\begin{array}{cc} 1 & \left(\left(v_{i},v_{j}\right)\in E,i\neq j\right)\\ 0 & (Others) \end{array}\right.$$
(2) Graph Convolution Module. The positioning data features of neighboring positioning points are extracted using the graph convolution module. The module includes sequentially connected graph convolution layers and fully connected layers. Input the node feature matrix ***X*** and the adjacency matrix ***A*** of the whole graph through a two-layer GCN to get the node-embedding matrix ***E***:
  (3) $$E=ELU[\hat{A}ELU(\hat{A}XW^{0})W^{1}]$$
  where $\hat{A}={\tilde{D}}^{-\frac{1}{2}}\tilde{A}{\tilde{D}}^{-\frac{1}{2}}$ is the normalized result of $I_{N}+D^{-\frac{1}{2}}AD^{-\frac{1}{2}}$, ***I_N_*** is the unit matrix, and ***D*** is the degree matrix of the graph. ***W***^0^ and ***W***^1^ denote the trainable weight matrices, and ELU is the activation function used in training the graph convolutional neural network. Therefore, the GCN outputs the prediction result $\hat{Y}$, which is represented as follows:
  (4) $$\hat{Y}=\omega ELU[\hat{A}ELU(\hat{A}XW^{0})W^{1}]+b$$
  where *ω* denotes the weight of the fully connected layer, and *b* denotes the bias term of the fully connected layer. They are both trainable parameters in neural networks.
(3) Output Module. Outputs the estimated position of the target.

### 2.3. Positioning Methods and Process Based on a GCN

**Figure 4:**
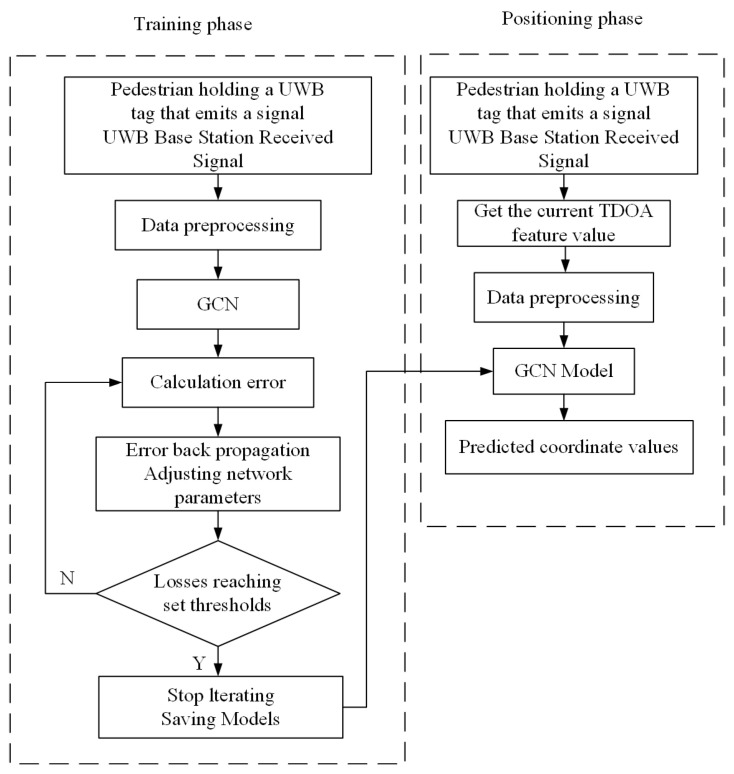
A block diagram of the UWB positioning method based on a GCN.

The block diagram of the positioning process is shown in Figure 4.

The UWB positioning method based on a graph convolutional neural network consists of the following four parts:

(1) Data acquisition. In the indoor environment where the UWB positioning system is installed, pedestrians walk around holding UWB tags that emit signals. The UWB base station receives the pulse signals from the tags. Then, it transmits the raw TDOA positioning data to the host computer through a switch, which serves as the a priori data for training the graph convolutional neural network.
(2) Training model. Firstly, we preprocess the base-station coordinates measured by the laser range finder and the raw TDOA positioning data received by the host computer. Secondly, these positioning data are constructed into graph data that conformed to the inputs of the graph convolutional neural network, including the feature matrix X and the graph’s adjacency matrix A. The difference between the predicted result $\hat{Y}$ output by the graph neural network and the actual label Y is compared. The deviation between them is calculated using the MSE loss function, and the gradient of the weights is calculated based on the loss function. Train the network by stochastic gradient descent and update the weight parameters of the network. The training is stopped until the loss is lower than the expected threshold. Finally, the model parameters are saved to get the optimal graph convolutional neural network model for UWB positioning.
(3) Model application. When pedestrians need location services, the pedestrian TDOA data and the base-station coordinates are input to the trained graph convolutional neural network model.
(4) Coordinate calculation. The graphical convolutional neural network model outputs a prediction of the pedestrian’s coordinates based on the positioning data input.

## 3. Visual Target Detection and Positioning

In indoor positioning systems, visual target detection is critical for supplementing UWB signals, especially in environments with obstacles or non-line-of-sight conditions. However, visual-based methods face challenges such as lighting variations, camera distortions, and occlusions, which can reduce positioning accuracy.

To overcome these limitations, we propose a visual localization method using a Bi-GRU network. This network learns residuals from visual positioning errors and corrects them in real time, improving accuracy by compensating for camera distortions and ensuring smoother pedestrian tracking. This approach enhances the reliability and continuity of visual-based positioning.

### 3.1. Video Image Pedestrian Detection

Detecting pedestrians in video imagery forms the fundamental component of visual positioning systems. The YOLOv7 algorithm has excellent detection accuracy, which is well applied to indoor positioning scenarios. Therefore, we used the YOLOv7 target-detection algorithm for pedestrian detection and recognition in video images.

**Figure 5:**
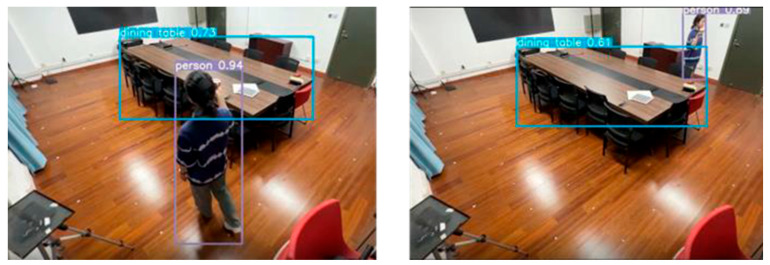
The YOLOv7 has excellent target-detection capabilities. It still works when the pedestrian is obscured by an obstacle.

Figure 5 shows the pedestrian detection results in video images based on the YOLOv7 algorithm. When a pedestrian appears on the surveillance screen, they are framed by a rectangular box that shows the target category and its confidence level. At the same time, the coordinates of the pedestrian’s plantar pixels calculated from the bounding box information are output.

### 3.2. Principle of Coordinate Frame Transformation

**Figure 6:**
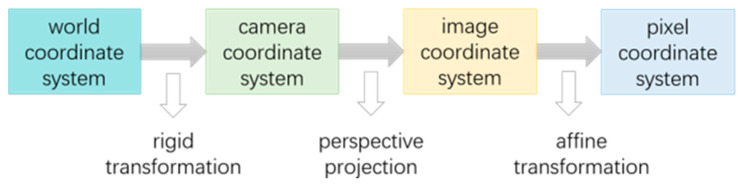
Principle of transformation between four coordinate systems.

The result obtained using the YOLOv7 target-detection algorithm is the pixel coordinates of the pedestrian. However, we need world coordinates. Converting pixel coordinates to world coordinates involves four coordinate systems: a pixel coordinate system, an image coordinate system, a camera coordinate system, and a world coordinate system. The conversion relationship between the coordinate systems is shown in Figure 6.

**Figure 7:**
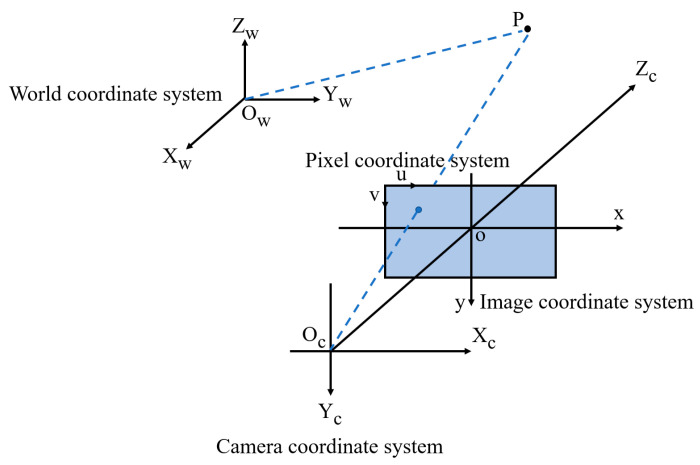
P-point coordinate transformation.

We select a point P in the 3D real world as an object, as shown in Figure 7. Its coordinate is (u,v) in the pixel coordinate system, (x,y) in the image coordinate system, $\left(X_{c},Y_{c},Z_{c}\right)$ in the camera coordinate system, and $\left(X_{w},Y_{w},Z_{w}\right)$ in the world coordinate system.

The transformation from the camera coordinate system to the world coordinate system is accomplished by a rigid body transformation. The conversion formula is as follows:

(5) $$\left[\begin{array}{c} X_{c}\\ Y_{c}\\ Z_{c} \end{array}\right]=R\left[\begin{array}{c} X_{w}\\ Y_{w}\\ Z_{w} \end{array}\right]+T$$

where ***R*** is a 3 × 3 rotation matrix, and ***T*** is a 3 × 1 translation matrix. To convert it to the form of homogeneous coordinates:

(6) $$\left[\begin{array}{c} X_{c}\\ Y_{c}\\ Z_{c}\\ 1 \end{array}\right]=\left[\begin{array}{cc} R & T\\ 0 & 1 \end{array}\right]\left[\begin{array}{c} X_{w}\\ Y_{w}\\ Z_{w}\\ 1 \end{array}\right]$$

In the above equation, the rotation matrix ***R*** can also be expressed as:

(7) $$R=\left[\begin{array}{ccc} 1 & 0 & 0\\ 0 & cos\alpha & sin\\ 0 & -sin & cos \end{array}\right]\left[\begin{array}{ccc} cos\beta & 0 & -sin\beta \\ 0 & 1 & 0\\ sin\beta & 0 & cos\beta \end{array}\right]\left[\begin{array}{ccc} cos\gamma & sin\gamma & 0\\ -sin\gamma & cos\gamma & 0\\ 0 & 0 & 1 \end{array}\right]$$

The conversion process from the ideal image coordinate system to the camera coordinate system is a 2D to 3D conversion, also known as perspective projection. When the focal length f of the camera is known, the conversion can be performed by the following equation:

(8) $$\left\{\begin{array}{c} x=f*\frac{X_{c}}{Z_{c}}\\ y=f*\frac{Y_{c}}{Z_{c}} \end{array}\right.$$

To convert it to the form of homogeneous coordinates:

(9) $$Z_{c}\left[\begin{array}{c} x\\ y\\ 1 \end{array}\right]=\left[\begin{array}{cccc} f & 0 & 0 & 0\\ 0 & f & 0 & 0\\ 0 & 0 & 1 & 0 \end{array}\right]\left[\begin{array}{c} X_{c}\\ Y_{c}\\ Z_{c}\\ 1 \end{array}\right]$$

In the above equation, *f* is the focal length of the camera.

The transformation of the pixel coordinate system to the image coordinate system undergoes an affine transformation. The mathematical expression is given below:

(10) $$\left\{\begin{array}{c} u=\frac{x}{dx}+u_{0}\\ v=\frac{y}{dy}+v_{0} \end{array}\right.$$

To convert it to the form of homogeneous coordinates:

(11) $$\left[\begin{array}{c} u\\ v\\ 1 \end{array}\right]=\left[\begin{array}{ccc} \frac{1}{dx} & 0 & u_{0}\\ 0 & \frac{1}{dy} & v_{0}\\ 0 & 0 & 1 \end{array}\right]\left[\begin{array}{c} x\\ y\\ 1 \end{array}\right]$$

where $\left(u_{0},v_{0}\right)$ represents the coordinates of the origin of the image coordinate system under the pixel coordinate system, and *dx* and *dy* denote the length of each pixel along the *x* and *y* axis, respectively, in millimeters.

To conclude, the relational equation for the conversion of pixel coordinates to world coordinates is as follows:

(12) $$Z_{c}\left[\begin{array}{c} u\\ v\\ 1 \end{array}\right]=\left[\begin{array}{cccc} \frac{f}{dx} & 0 & u_{0} & 0\\ 0 & \frac{f}{dy} & v_{0} & 0\\ 0 & 0 & 1 & 0 \end{array}\right]\left[\begin{array}{cc} R & T\\ 0^{T} & 1 \end{array}\right]\left[\begin{array}{c} X_{w}\\ Y_{w}\\ Z_{w}\\ 1 \end{array}\right]$$

where $\left(X_{w},Y_{w},Z_{w}\right)$ represents the world coordinates corresponding to the pixel coordinates (u,v). *f*, *dx*, *dy*, *u*_0_, and *v*_0_ are determined internally by the camera and are called camera internal parameters. Moreover, ***R*** and ***T*** are determined by the relative position between the camera and the world coordinate system and are called camera external references.

This work uses the Zhang Zhengyou camera calibration method [25] to obtain the camera’s internal parameters. The camera’s external parameters are calculated from the position of the origin of the world coordinate system. The results of the initial positioning of pedestrians in world coordinates can be obtained using the coordinate transformation formula. Since the camera parameters are known, it is possible to calculate the initial positioning results of pedestrians in world coordinates. However, Zhang’s method only focuses on the radial distortion in the camera distortion without considering the tangential distortion. There are still positioning errors due to camera distortion. Therefore, this paper will follow up with further research on improving the error caused by camera distortion.

### 3.3. Indoor Visual Positioning Method Based on Bi-GRU and Residual Fitting

There are two main types of ideas for camera calibration: traditional physical model-based approaches and data-driven-type approaches. Zhang’s method mentioned above is the classical physical model-based camera calibration method. It is based on a specific geometric model with a precise mathematical form, and the model parameters have actual physical significance. However, this method only considers the more influential radial distortion. With the rapid development of artificial intelligence technology, data-driven camera calibration models are beginning to emerge. This type of model attempts to learn the mapping relationships of coordinate transformations from a large amount of end-to-end paired data. The nonlinear fitting ability of neural networks implicitly represents the internal and external parameters of the camera. It does not have to calibrate the image and has excellent accuracy. However, the parameters in these “black box” technologies have no physical meaning and are poorly interpretable. Their outputs are difficult to control in real-world scenarios. To integrate the advantages of the two methods, this paper uses residual fitting to compensate for the error in the physical-class model results using a Bi-GRU network to remedy the visual positioning errors generated by camera aberrations.

**Figure 8:**
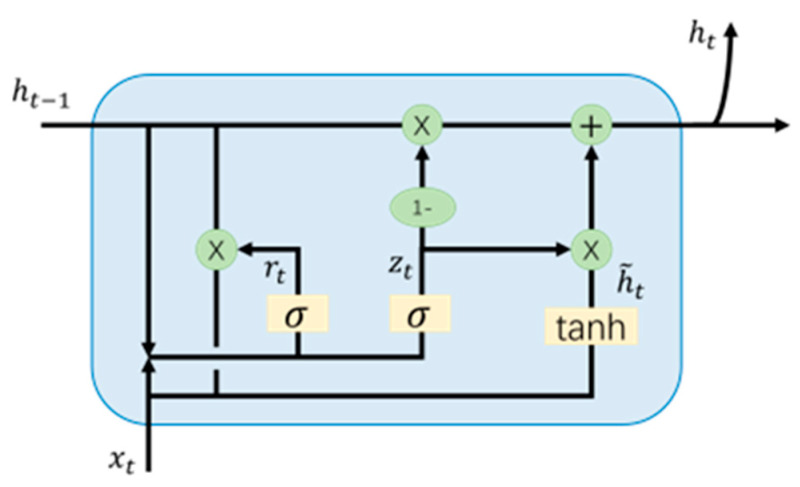
Structure of a single GRU neuron.

As we all know, a recurrent neural network (RNN) is a class of neural networks with memory capability. However, the disadvantage is that it cannot solve the long-term dependence. The extended short-term memory network (LSTM) introduces input, forgetting, and output gates. It can capture long-time dependencies but has the disadvantage of complex structure and inefficient training. Compared to LSTM, GRU has only two gating units, an update gate and a reset gate, with fewer parameters. The structure of a GRU neuron is shown in Figure 8.

The specific data transfer process can be represented as follows:

(13) $$r_{t}=\sigma (W_{rx}x_{t}+h_{t-1}W_{rh}+b_{r})$$

(14) $$z_{t}=\sigma (W_{zx}x_{t}+h_{t-1}W_{zh}+b_{z})$$

(15) $${\tilde{h}}_{t}=\tan h(W_{hx}x_{t}+r_{t}⊙h_{t-1}W_{hh}+b_{h})$$

(16) $$h_{t}=(1-z_{t})⊙h_{t-1}+z_{t}⊙{\tilde{h}}_{t}$$

where *x_t_* denotes the current input, and σ and tan*h* are activation functions that compress the output into the range 0 to 1. *W_rx_*, *W_rh_*, *W_zx_*, *W_zh_*, *W_hx_*, and *W_hh_* are the weights to be updated, ⊙represents the dot-multiplication operation, and ${\tilde{h}}_{t}$ and $h_{t}$ are candidate hidden states and hidden states, respectively.

**Figure 9:**
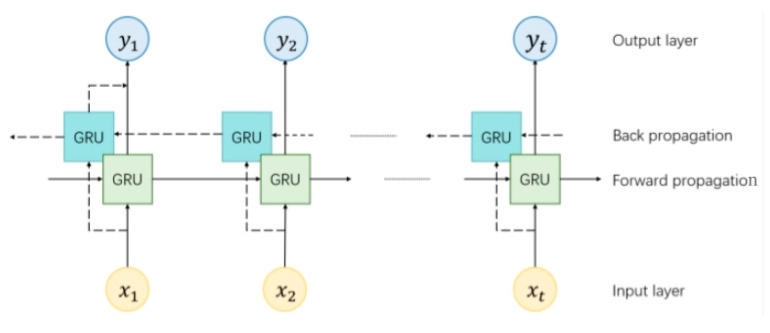
Bi-GRU network model.

The above standard GRU only extracts sequence features in a single direction, whereas the Bi-GRU has an additional GRU with input in the opposite direction. By training both forward and reverse simultaneously, the complete contextual information of the input sequence can be captured. By applying bi-directional GRU to positioning, historical and future time-series positioning data are used to provide feedback to correct the model parameters while training the network. The bi-directional GRU network provides historical position and orientation information for position prediction at the current moment, thus outputting a more accurate position. The principle of Bi-GRU is shown in Figure 9.

**Figure 10:**
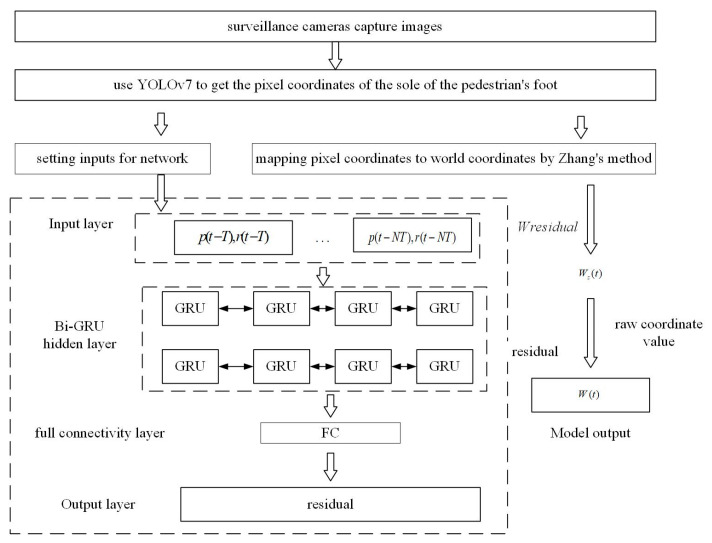
Compensate the coordinate residuals learned by the Bi-GRU network into the initial coordinates computed by Zhang’s method.

This article proposes a framework for an indoor visual positioning model based on residual fitting. A residual exists between the raw values of the world coordinate obtained by Zhang’s method and the actual values. A Bi-GRU network fits this residual, and the compensated value is added to the basic coordinate to get the pedestrian’s more accurate world coordinate value. The modeling framework is shown in Figure 10.

In this article, the input to the Bi-GRU network model is the information extracted from the past ten surveillance frames, including the pixel coordinates and the residuals of the original and actual values of the coordinates. Ultimately, the model can be expressed by the following equation:

(17) $$W(t)=W_{z}(t)+W_{r}(t)$$

(18) $$W_{r}(t)=f\left(\begin{array}{c} p(t-T),r(t-T),\\ p(t-2T),r(t-3T),\\ \ldots \\ p(t-NT),r(t-NT) \end{array}\right)$$

where *W*(t) represents the predicted pedestrian coordinates output from the model. *W_z_*(t) denotes the raw value of pedestrian coordinates computed from the parameters obtained through Zhang’s method. *W_r_*(t) represents the residuals between the actual world coordinates and the initial coordinates computed by Zhang’s camera calibration method, and *f*(.) represents the coordinate residual fitting function learned by the Bi-GRU network. N = 10 denotes the memory length of the input sequence. T is the length of one video frame, and *p*(*t* − *kT*) and *r*(*t* − *kT*) are denoted as the pedestrian pixel coordinates and coordinate residuals output by the target-detection algorithm at the moment (*t* − *kT*), respectively.

## 4. Fusion Positioning

Filtering is employed for fusion to integrate UWB and visual data. Currently, the most widely used data-fusion methods include the extended Kalman filter (EKF) and the cubature Kalman filter (CKF). Although EKF is renowned for its simplicity in implementation, it suffers from model errors introduced during linearization, hindering further improvements in positioning accuracy. CKF exhibits greater linearization accuracy when addressing nonlinear issues. However, as the complexity and dimensionality of the system increase, CKF stability becomes challenged, leading to performance degradation or even collapse. Leveraging the law of large numbers for integral computations, PF has emerged as a potent tool for addressing nonlinear-system positioning issues. With powerful nonlinear filtering capabilities, PF can achieve near-optimal system state estimation, offering a new solution for enhancing positioning accuracy and stability.

### 4.1. Particle Filter Algorithm

The core principle of the particle filter entails representing system states via stochastic variables. Each filtering step approximates the probability distribution of the system state using discrete random variables and employs the sample mean of these random variables instead of the integral value. The theoretical foundation of the particle filter involves treating the system state as a random variable, given its probability density function *p*(*x*), constructing an importance density function *q*(*x*) related to the system state, and deriving the importance weight *w*(*x*) of the system state:

(19) $$w(x)=\frac{p(x)}{q(x)}$$

Given the measurement values $z_{1:k}=\left\{z_{i},i=1,\cdots ,k\right\}$ for the system at this moment, and $x_{0:k}=\{x_{i},i=1,\cdots ,k\}$ representing the corresponding system states, a set of particles $q(x_{0:k}|z_{1:k})$, weighted by $\left\{w_{k}^{i},i=0,\cdots ,N\right\}$, is sampled from the significance density function $\left\{x_{0:k}^{i},i=0,\cdots ,N\right\}$. This approximates the posterior probability distribution $p(x_{0:k}|z_{1:k})$ of the system state at time *k*. In other words, the posterior probability distribution of the system state at time *k* can be described as:

(20) $$p(x_{0:k}|z_{1:k})\approx \sum_{i=1}^{N}w_{k}^{i}\delta (x_{0:k}-x_{0:k}^{i})$$

(21) $$w_{k}^{i}\propto \frac{p(x_{0:k}^{i}|z_{1:k})}{q(x_{0:k}^{i}|z_{1:k})}$$

In the formula, *N* represents the number of sampled particles, *δ* denotes the Dirac function, $w_{k}^{i}$ denotes the weight of particle $x_{0:k}^{i}$, and $\sum_{i}w_{k}^{i}=1$. The choice of importance density function *q*(*x*) is a primary factor influencing the performance of particle filtering and serves as a criterion for distinguishing among different particle filters. This paper chooses the prior probability density, which is the easiest to implement, as the critical density function, as depicted in Formula (24):

(22) $$q(x_{k}^{i}|x_{k-1}^{i},z_{k})=p(x_{k}^{i}|x_{k-1}^{i})$$

Based on the prediction process of Bayesian inference, the posterior probability distribution is recursively updated:

(23) $$p(x_{0:k}|z_{1:k})\propto p(z_{k}|x_{k})p(x_{k}|x_{k-1})p(x_{0:k-1}|z_{1:k-1})$$

By substituting Equations (17) and (18) into Equation (19), we obtain the updated Formula (20), as follows:

(24) $$w_{k}^{i}\propto w_{k-1}^{i}p(z_{k}|x_{k}^{i})$$

Normalize weight $w_{k}^{i}$ is calculated as follows:

(25) $$w_{k}^{i}=\frac{w_{k}^{i}}{\Sigma_{i=1}^{N}w_{k}^{i}}$$

The posterior probability density $p(x_{k}|z_{1:k})$ can be represented as follows:

(26) $$p(x_{k}|z_{1:k})\approx \sum_{i=1}^{N}w_{k}^{i}\delta (x_{k}-x_{k}^{i})$$

As depicted in Equation (26), as the particle count *N*→∞, the right-hand side progressively approximates the actual posterior probability $p(x_{k}|z_{1:k})$, as dictated by the law of large numbers.

### 4.2. UWB/Visual Data Fusion Algorithm Based on Particle Filtering

The indoor pedestrian positioning system discussed in this paper, which combines UWB and visual data, is a typical nonlinear stochastic system employing particle filtering as the fusion algorithm. Assuming the system state is represented by two-dimensional position information $X_{i}={\left[x_{i},y_{i}\right]}^{T}$, the UWB positioning value is designated as the system’s measurement $Z_{i}={\left[x_{i}^{z},y_{i}^{z}\right]}^{T}$, while visual positioning is represented by the particle transition model, as shown in Equation (29), which can be written in matrix form as follows:

(27) $$U_{i}=\left[\begin{array}{cc} 1+(L_{i}\sin\theta_{i})/x_{i-1} & 0\\ 0 & 1+(L_{i}\cos\theta_{i})/y_{i-1} \end{array}\right]$$

The steps for using PF fusion are as follows:

(1) Initialization

Consider the UWB initial positioning location $\left(x_{z},y_{z}\right)$ as the actual value. Generate a set of particles $\left\{x_{0}^{i},i=1,\cdots ,N\right\}$ and their corresponding weights $\left\{w_{0}^{i},i=1,\cdots ,N\right\}$ based on the Gaussian white noise model, setting $\sum_{i=1}^{N}w_{0}^{i}=1$.

(2) Update

After performing visual positioning at time *k*, update the position of the particle set from the previous time *k* − 1 using Equation (27), as follows:

(28) $$X_{k}^{i}=U_{i}X_{k-1}^{i}$$

At time *k*, the UWB positioning position may not have been updated, so interpolation is used to calculate the UWB positioning position $\left(x_{k}^{z},y_{k}^{z}\right)$ at time *k*. The updated Euclidean distance $d_{k}^{i}$ for UWB positioning is shown below:

(29) $$d_{k}^{i}=\sqrt{{\left(x_{k}^{i}-x_{k}^{z}\right)}^{2}+{\left(x_{k}^{i}-y_{k}^{z}\right)}^{2}}$$

Particle weights are updated based on Euclidean distance A, meaning the smaller the distance between particles and observations, the higher the weight. The updated formula is:

(30) $$w_{k}^{i}=\frac{{\left(d_{k}^{i}\right)}^{-1}}{\sum_{i=0}^{N}{\left(d_{k}^{i}\right)}^{-1}}$$

Re-normalize to ensure $\sum_{i=1}^{N}w_{k}^{i}=1$.

(3) Resampling

To prevent particle degradation, it is essential to resample particles based on their importance, aiming to eliminate those with lower weights. Initially, assess the extent of particle degradation:

(31) $$N_{eff}=\frac{1}{\sum_{i=1}^{N}{\left(w_{k}^{i}\right)}^{2}}$$

If $N_{eff}\leq N$, indicating a high degree of particle degradation, resampling is necessary. Resampling initially produces *N* sets of numbers $\mathbb{C}=\{\varepsilon_{i},i=1,\cdots ,N\}$, each following a uniform distribution within the range of [0, 1], and computes the cumulative particle probabilities:

(32) $$a_{j}=\sum_{i=1}^{j}w_{k}^{i}$$

Generate *N* cumulative probability values $\left\{a_{j},j=1,\cdots ,N\right\}$. It is known that $a_{j}$ constitutes an ascending sequence. Sequentially extract the ith(i=1,2,⋯,N) numerical value $\varepsilon_{i}$ from the dataset ℂ, and ascertain its corresponding cumulative probability interval, satisfying the condition:

(33) $$a_{j-1}\leq \varepsilon_{i}\leq a_{j}$$

Subsequently, the *i*th particle $X_{k}^{i}$ can be retrieved from the original particle set to serve as the new particle ${\dot{X}}_{k}^{i}$. Particles with greater weights in the original set are replicated multiple times, whereas those with lower weights are discarded. Lastly, normalize the weights of the new particles.

(4) Output

Compute the expected particle-set post-resampling, as shown below:

(34) $$\left\{\begin{array}{l} {\bar{x}}_{k}=\sum_{i=1}^{N}x_{k}^{i}w_{k}^{i}\\ {\bar{y}}_{k}=\frac{1}{n}\sum_{i=1}^{N}y_{k}^{i}w_{k}^{i} \end{array}\right.$$

The expectation of the particle set represents the optimal position for fusion positioning, with the PF fusion positioning result at time *N* being $\left({\bar{x}}_{k},{\bar{y}}_{k}\right)$. Await the subsequent visual positioning, then repeat the updating, resampling, and outputting steps.

## 5. Evaluation

**Figure 11:**
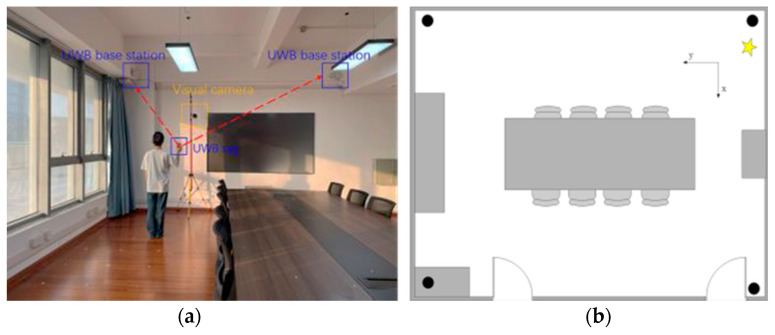
Images of the experimental site. (**a**): laboratory environment. (**b**): structure map of the laboratory environment.

The experimental site we built has an area of 7.5 m × 6.0 m, as shown in Figure 11a,b. We deploy four UWB stations and one camera in it. The positioning base station is connected to the host computer through a switch, while the visual camera transmits the surveillance video to the host computer for subsequent positioning. The lab site has more tables and chairs as well as a large expanse of glass, which is consistent with static interference conditions in a general indoor environment.

**Figure 12:**
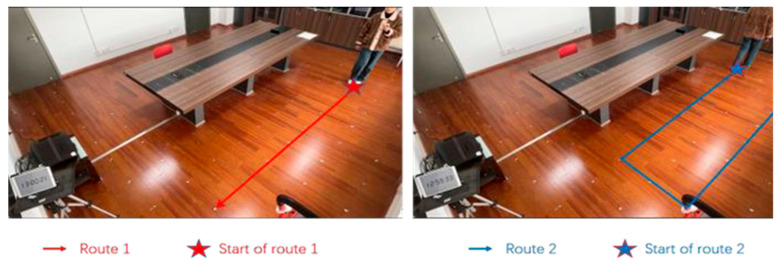
The two positioning routes selected for the experiment.

Two distinct experimental routes were established to substantiate the versatility of the proposed positioning methodology. The experimental routes are shown in Figure 12.

### 5.1. Experimental Results of the UWB Positioning

During the experiment, the UWB-positioning base station receives the positioning signal transmitted by the tag. The upper computer takes the TDOA raw positioning data as the input of the graph convolutional neural network, and the output data results from pedestrian positioning coordinates. At the same time, the original TDOA positioning data are substituted into the classic Chan–Taylor positioning algorithm for pedestrian coordinate settlement as a comparative experimental result.

**Figure 13:**
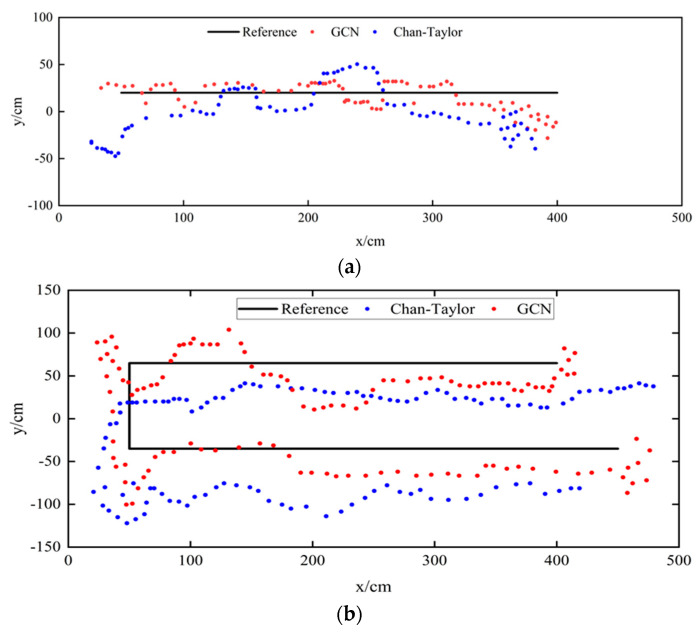
Comparison of the Chan–Taylor method and our proposed method for different route trajectories. (**a**): Route-1; (**b**): Route-2.

For Experiment Route 1 and Experiment Route 2, the actual trajectory of pedestrians, the pedestrian trajectory calculated by the Chan–Taylor positioning algorithm, and the positioning results of the UWB indoor pedestrian positioning model based on a graph convolutional neural network are shown in Figure 13.

**Figure 14:**
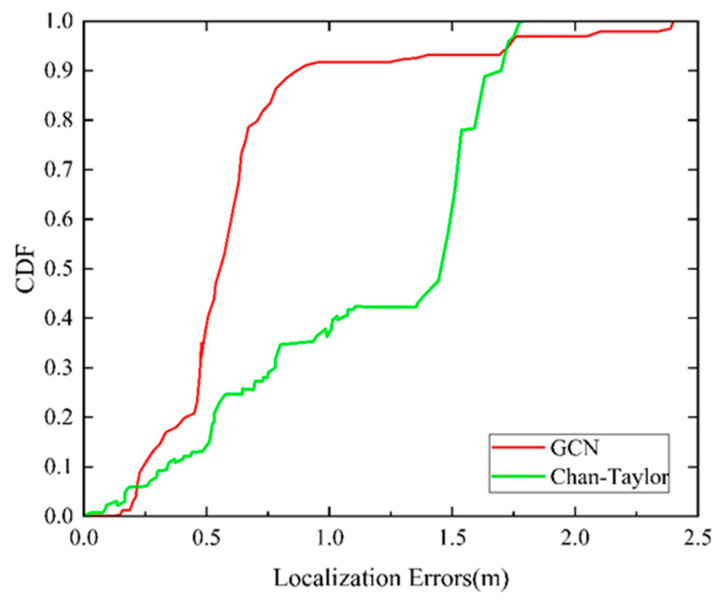
CDF of positioning errors of the Chan–Taylor method and our proposed method.

To be specific, Figure 14 shows that when CDF is 80%, the positioning accuracy of the UWB positioning method based on Chan–Taylor is about 1.54 m, and the maximum error can be more than 1.75 m. In the same way, the positioning accuracy of the UWB positioning method based on a GCN is less than 0.67 m. By comparison, 80% of the positioning errors of the optimization methods combine both within 0.64 m, and the maximum error is limited to 1.27 m, solving the problem of neural network models outputting outliers effectively. Intuitively, optimization methods based on the combination of a GCN and Chan–Taylor achieve high-quality performance in this experiment. This method enhances the robustness of positioning and improves indoor pedestrian positioning accuracy.

### 5.2. Experimental Results of the Visual Positioning

Accomplishing visual positioning begins with calibrating the camera. In this experiment, the internal and external parameters of the camera are calibrated using Zhang’s method. The calculated parameter values are shown in Table 1.

As a result, the pixel-to-world coordinate system conversion formula is determined. Videos of pedestrians walking indoors are captured using the camera installed at the experimental site, which serves as the a priori dataset for this experiment. We get the pixel coordinates of the pedestrian’s soles with YOLOv7. With the help of the coordinate system transformation formula, we obtain the raw world coordinates of the pedestrian.

The Bi-GRU residual fitting network inputs are pedestrian pixel coordinates and raw world coordinates. The difference between the actual world coordinate value of the pedestrian and the coarse value of the coordinate is set as the label. Then, the network can be trained. The parameters of the network model are shown in Table 2.

**Figure 15:**
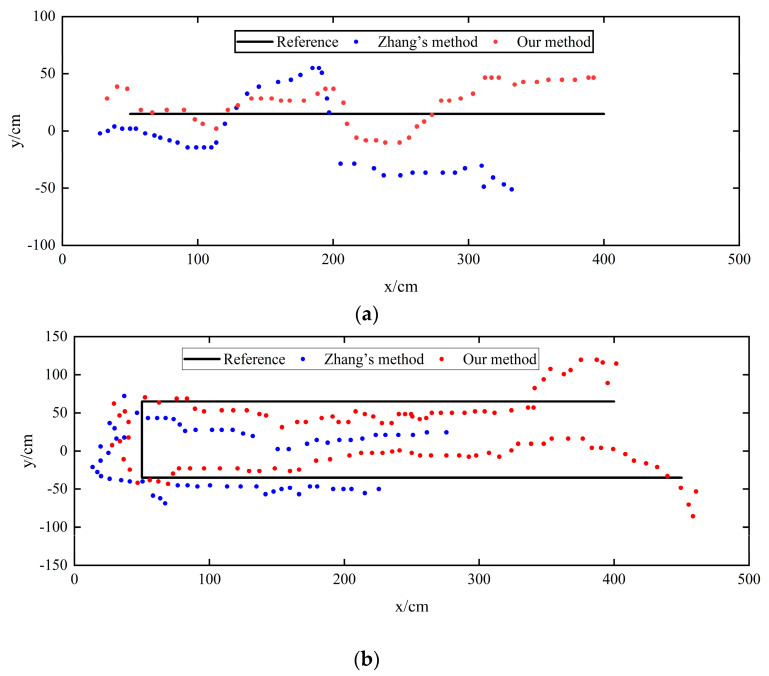
Comparison of Zhang’s and our proposed methods in different route trajectories. (**a**): Route-1; (**b**): Route-2.

Figure 15a,b show the positioning results for the two experimental routes and the actual trajectories. Figure 1 and Figure 2 illustrate that the initial visual positioning results fluctuate widely. This is because the camera is located at (−100 cm, 100 cm) in the coordinate system, indicating a distance from the positioning area. The farther away from the camera, the closer to the edges of the video frame, and the more pronounced the distortion effect becomes. The improved visual positioning method effectively corrects the world coordinates when the pedestrian is at the edge of the video frame.

**Figure 16:**
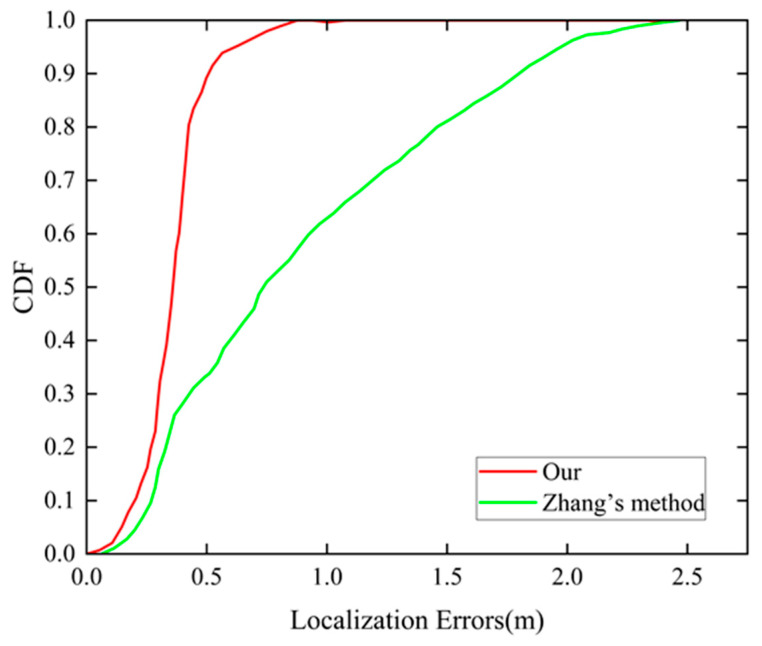
CDF of positioning errors of Zhang’s method and our proposed method.

In addition, we provide the CDF of visual errors in Figure 16 to better evaluate the positioning effectiveness of the proposed method. It can be intuitively seen that in the initial visual positioning results, the accuracy of 80% of the results is within 1.45 m, and the maximum error is even more than 2 m. In contrast, 80% of the improved visual positioning results are within 0.42 m, and the maximum error is narrowed down to 0.89 m, and the positioning accuracy is improved by 71%. It appears that the method we propose effectively improves the visual positioning error due to lens aberration with more robustness and less error fluctuation.

### 5.3. Experimental Results of the Fusion Positioning

Firstly, we weighted the fusion of pedestrian coordinates from two sensors to accomplish fusion positioning.

In terms of complex indoor environments, the effects of NLOS and multipath prevent UWB systems from providing continuous and stable positioning signals, resulting in positioning interruptions. Thus, we propose a data fusion method. Specifically, when the UWB signal is interrupted and positioning is unavailable, we extend the results of the previous moment to the current moment. Next, we fuse it with the contemporary visual positioning result as the observation of the particle filter. On the other hand, when the two positioning methods output coordinates simultaneously, they are fused directly to produce one observation.

**Figure 17:**
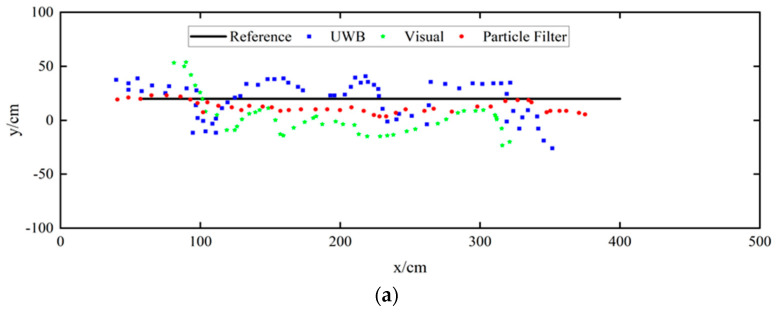

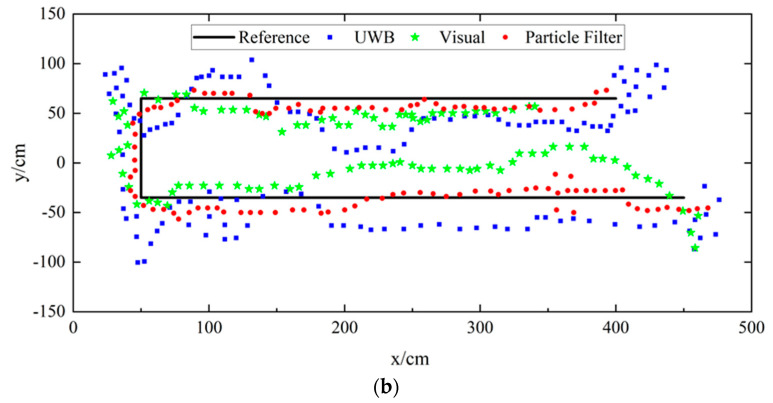
Comparison of the UWB method, visual method, and our proposed method in different route trajectories. (**a**): Route-1; (**b**): Route-2.

Compared with UWB or visual positioning individually, the particle-filtered pedestrian trajectories are closer to the actual movement routes. This situation can also be observed in Figure 17. We can also notice that the positioning points obtained by the UWB positioning technique are discrete. The reason is that the UWB signal is affected by the human-body occlusion and multipath effect, causing insufficient observations of the original TDOA data. Besides, the single visual positioning suffers from low positioning accuracy. However, after data fusion and particle filtering, the continuity of visual positioning partially compensates for the missing part of UWB positioning. At the same time, a few UWB positioning results with higher accuracy also improve the accuracy of visual positioning.

**Figure 18:**
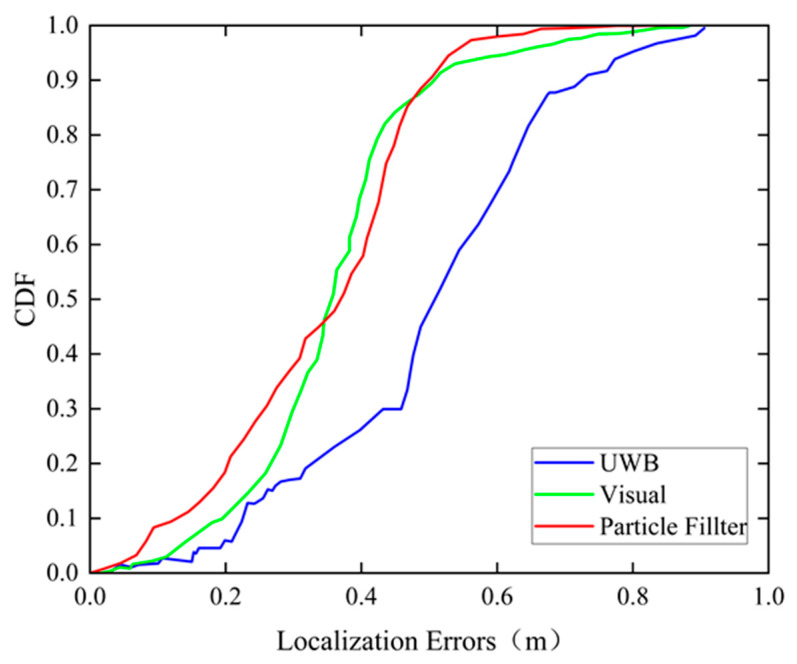
CDF of positioning errors of the UWB method, visual method, and our proposed method.

To show the fusion algorithm intuitively, we give the CDF curves of the positioning errors of the three methods proposed in Figure 18. It can be seen that the visual and fusion positioning techniques have similar positioning accuracies in most cases, with 90% of the positioning points holding an accuracy within 0.49 m. However, compared to the maximum error of 0.88 m in the visual positioning result, it is reduced to 0.77 m after fusion filtering, which improves the positioning accuracy by 12.5%.

## 6. Conclusions

This paper presents an indoor pedestrian positioning approach that leverages the fusion of UWB and visual positioning, employing particle filtering techniques for enhanced accuracy. In this method, we fuse the advantages of the two positioning methods. On the one hand, visual positioning’s high stability and continuity improve the problem of shaky positioning data in UWB applications. On the other hand, UWB positioning reduces the errors caused by lens distortion in visual positioning. We have also proposed improved approaches in individual UWB and visual positioning techniques to exert a better fusion positioning effect. Among them, the UWB indoor pedestrian positioning method based on GCN fully uses the feature information of neighboring points. It effectively overcomes the interference of complex indoor environments on positioning. An indoor visual positioning model based on bi-directional GRU with residual fitting integrates the strengths of both physical models and neural networks, somewhat reducing the positioning error due to camera distortion. The advantages of the proposed method are also suggested through real-world experiments. In future work, we will consider exploring better multi-sensor fusion methods to maximize the benefits of integrating single positioning techniques. In addition, the above methods are expected to be applied to more complex indoor scenes, such as visual blind-area positioning and seamless indoor–outdoor positioning.

## Figures and Tables

**Table 1:** Camera parameters.

Parameter Types	Symbol	Value
Internal Parameters	*f_x_*	747.3596
	*F_y_*	747.4950
	*u*_0_	637.8703
	*v*_0_	360.3958
External Parameters	**R**	$\left[\begin{array}{ccc} 0.7285 & -0.4492 & 0.5173\\ 0.6846 & 0.4503 & -0.5732\\ 0.0245 & 0.7717 & 0.6355 \end{array}\right]$
	T	$\left[\begin{array}{c} 29.1905\\ 1028.33082\\ 2697.8627 \end{array}\right]$

**Table 2:** Network model parameter configuration table.

Parameter Type Attributes	Attributes
Input Vector Dimension	40
Output Vector Dimension	2
The Number of Hidden Layer Nodes	256
The Number of Hidden Layers	2
Loss Function	MSE
Learning Rate	0.00001
Optimizer	Adam
Training Batches	50

## Data Availability

The data that support the findings of this study are available from the first and corresponding author upon reasonable request.
